# Additive manufacturing of LiNi_1/3_Mn_1/3_Co_1/3_O_2_ battery electrode material via vat photopolymerization precursor approach

**DOI:** 10.1038/s41598-022-22444-1

**Published:** 2022-11-08

**Authors:** Ana C. Martinez, Alexis Maurel, Ana P. Aranzola, Sylvie Grugeon, Stéphane Panier, Loic Dupont, Jose A. Hernandez-Viezcas, Bhargavi Mummareddy, Beth L. Armstrong, Pedro Cortes, Sreeprasad T. Sreenivasan, Eric MacDonald

**Affiliations:** 1 Department of Aerospace and Mechanical Engineering, The University of Texas at El Paso, El Paso, TX 79968 USA; 2 Laboratoire de Réactivité et de Chimie des Solides, UMR CNRS 7314, Hub de l’Énergie, Université de Picardie Jules Verne, 80039 Amiens Cedex, France; 3 RS2E, Réseau Français sur le Stockage Électrochimique de l’Energie, FR CNRS 3459, 80039 Amiens, France; 4 Laboratoire des Technologies Innovantes, LTI-EA 3899, Université de Picardie Jules Verne, 80025 Amiens, France; 5 Department of Chemistry and Biochemistry, The University of Texas at El Paso, El Paso, TX 79968 USA; 6 Department of Civil, Environmental, and Chemical Engineering, Youngstown State University, Youngstown, OH 44555 USA; 7 Materials Science and Technology Division, Oak Ridge National Laboratory, Oak Ridge, TN 37831 USA; 8 Manufacturing Science Division, Oak Ridge National Laboratory, Oak Ridge, TN 37830 USA

**Keywords:** Chemistry, Energy science and technology, Engineering, Materials science

## Abstract

Additive manufacturing, also called 3D printing, has the potential to enable the development of flexible, wearable and customizable batteries of any shape, maximizing energy storage while also reducing dead-weight and volume. In this work, for the first time, three-dimensional complex electrode structures of high-energy density LiNi_1/3_Mn_1/3_Co_1/3_O_2_ (NMC 111) material are developed by means of a vat photopolymerization (VPP) process combined with an innovative precursor approach. This innovative approach involves the solubilization of metal precursor salts into a UV-photopolymerizable resin, so that detrimental light scattering and increased viscosity are minimized, followed by the in-situ synthesis of NMC 111 during thermal post-processing of the printed item. The absence of solid particles within the initial resin allows the production of smaller printed features that are crucial for 3D battery design. The formulation of the UV-photopolymerizable composite resin and 3D printing of complex structures, followed by an optimization of the thermal post-processing yielding NMC 111 is thoroughly described in this study. Based on these results, this work addresses one of the key aspects for 3D printed batteries via a precursor approach: the need for a compromise between electrochemical and mechanical performance in order to obtain fully functional 3D printed electrodes. In addition, it discusses the gaps that limit the multi-material 3D printing of batteries via the VPP process.

## Introduction

Driven by the growing demand for portable consumer electronics and electric vehicles, in-depth research efforts have been devoted in the past years to improving energy and power density of lithium-ion batteries^1,2^. Compared to conventional parallel-plate (2D) battery configuration, three-dimensional (3D) battery architectures can exhibit enhanced electrochemical performances due to their greater electroactive surface area, and an improved lithium ion diffusion^3,4^. Consequently, the 3D batteries have shown a superior specific capacity, areal energy, and power density than traditional 2D batteries. Based on these promising aspects, the development of nanorods and post arrays as a 3D independent electrode has been achieved by electrochemical growth onto a substrate, followed by electrophoretic deposition of the battery electroactive material^5–7^. Unfortunately, the intercalation of independent 3D electrodes has often resulted in short circuits caused by the numerous surface irregularities.

Recent research in this area has made significant advances by utilizing 3D printing, owing to its ability to build intricate and tailored shapes with high resolution^8–11^. During the VPP process, a photopolymerizable resin is selectively polymerized to a crosslinked layer upon UV light exposure, and the procedure is repeated to create a macrostructure layer after layer with features down to 100 nm^12^. This technique has been employed to 3D print composite resins highly loaded with ceramic particles of Al_2_O_3_^13^, ZrO_2_^14^, and SiO_2_^15–17^. Since battery materials often consist of oxide compounds, their direct addition to the resin followed by 3D printing of battery electrodes is thus conceivable. However, it presents two critical challenges: (1) the increase in viscosity of the composite resin and (2) the detrimental light scattering caused by the presence of solid particles in the resin, increasing the difficulty of printing. This work uses VPP printing combined with a precursor approach to circumvent these problems. The process involves the addition of soluble precursor salts in stoichiometric amounts into a classical photopolymerizable resin, followed by in-situ synthesis of the material upon thermal post-processing of the printed item^12^. This approach presents the advantage of free-flowing resins with low viscosity, and no detrimental UV light-scattering as the soluble precursor salts are completely mixed at the molecular level in the resin. The photopolymerization and printing are consequently more efficient and accurate in comparison with conventional powder-loaded resins. Focused on the precursor approach, Fu et al.^18^ reported the preparation of an acrylate-based resin loaded with zirconium n-propoxide as a precursor. After sintering of the 3D printed structures up to 1200 °C, the authors successfully demonstrated the preparation of ZrOC 3D structures^18^. In 2021, Yee et al.^19^ reported the preparation of an aqueous acrylate-based resin containing Co(NO_3_)_2_.6H_2_O and LiNO_3_ in stoichiometric ratio. After calcination at 700 °C of the 3D printed structure, the authors demonstrated the in-situ synthesis of a LiCoO_2_ lithium-ion battery positive electrode material. Tested in half-cell configuration versus lithium metal, a capacity of 121 mAh g^−1^ was obtained at C/40, while the capacity retention was 76% after 100 cycles at C/10.

This work demonstrates for the first time the 3D printing of a lithiated ternary oxide with formula LiNi_1/3_Mn_1/3_Co_1/3_O_2_ (NMC 111) using the precursor approach. The synthesis of an NMC material following the precursor technique has been selected mainly due to the enormous commercial interest in high energy density NMC materials from the automobile industry^20,21^. Moreover, 3D printing can be particularly useful for a wide range of applications including electric cars as it could potentially allow the production of shape-conformable energy storage devices^22^. Among the diverse NMC materials, the 111 composition was selected because of its reduced reactivity in air^23,24^, and the inexistence of Ni^3+^, thus minimizing Li^+^-Ni^3+^ cation mixing during in-situ synthesis^25,26^. Herein, the development of a composite photocurable resin, loaded with soluble lithium, nickel, manganese and cobalt precursor salts in stoichiometric ratio is described. After the VPP printing step, the shape-conformable 3D printed items containing the precursors are subjected to a thermal treatment where in-situ synthesis of NMC 111 occurs. Thermogravimetric analysis (TGA) of the composite resin, as well as structural, elemental, morphological and electrochemical analysis of the electrode materials in a half-cell lithium-ion battery configuration is reported. Thermal treatment parameters are optimized with a view to obtain free-standing NMC 111 3D structures with the highest possible electrochemical performance.

## Results and discussion

### Manufacturing process

**Figure 1:**
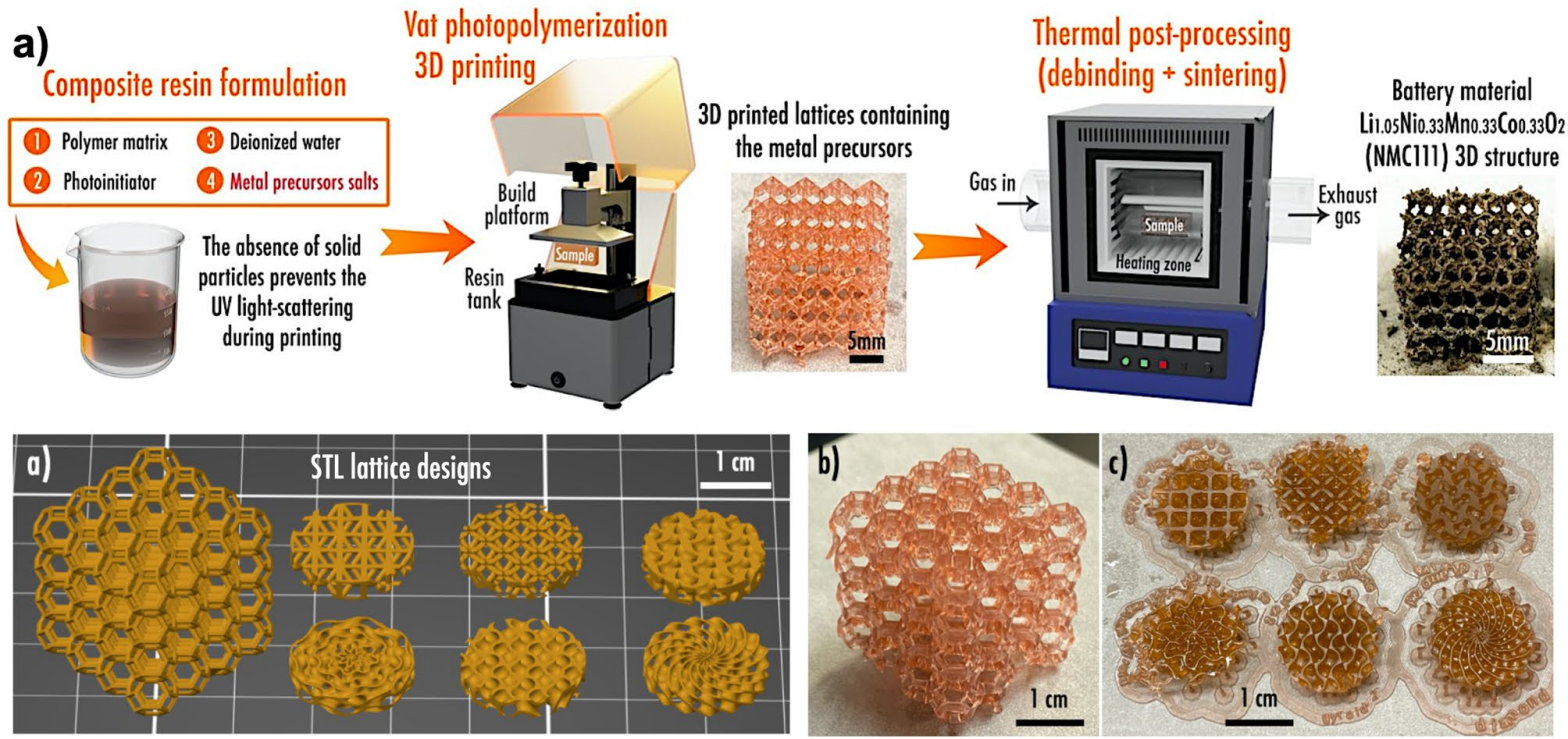
(**a**) Main steps of the NMC 111 3D structures fabrication process; (**b**) Standard Tessellation Language (STL) numerical models of the complex truncated octahedron and disc lattices with various infill patterns printed in this work; (**c**) actual 3D printed truncated octahedron and (**d**) discs, containing NMC 111 precursors.

The complete manufacturing process to obtain NMC 111 3D structures is displayed in Fig. 1a and consists of (1) composite resin formulation, (2) VPP 3D printing, and (3) thermal post-processing. First, a composite UV photopolymerizable resin was prepared by mixing the aqueous solution of metal precursor salts in stoichiometric amounts, photoinitiator, and polymer matrix; as described in the *Materials and methods section*. Nitrate precursor salts were selected as the metal precursors among many different salts due to their superior solubility in water^27,28^. The higher solubility of salts also enabled the preparation of comparatively concentrated metal solutions (3 mol L^−1^). The second step consisted of printing the composite resin. Due to the incorporation of soluble precursors in the resin, the printing step was more efficient in comparison with conventional ceramic powder-loaded resins, as no detrimental UV light scattering or other sedimentation issues were observed. Hence, by locking the geometry with additive manufacturing, different complex 3D structures were manufactured, including a cube composed of a truncated octahedron lattice and discs with intricate infill patterns (Fig. 1b-d). Finally, the third step consisted of the thermal post-processing of printed pieces. During the thermal post-processing, the non-electroactive polymer matrix and photoinitiator were removed (debinding step), and the electroactive NMC 111 material was synthesized and sintered.

### Thermal characterization of the resins and synthesis of the NMC material

**Figure 2:**
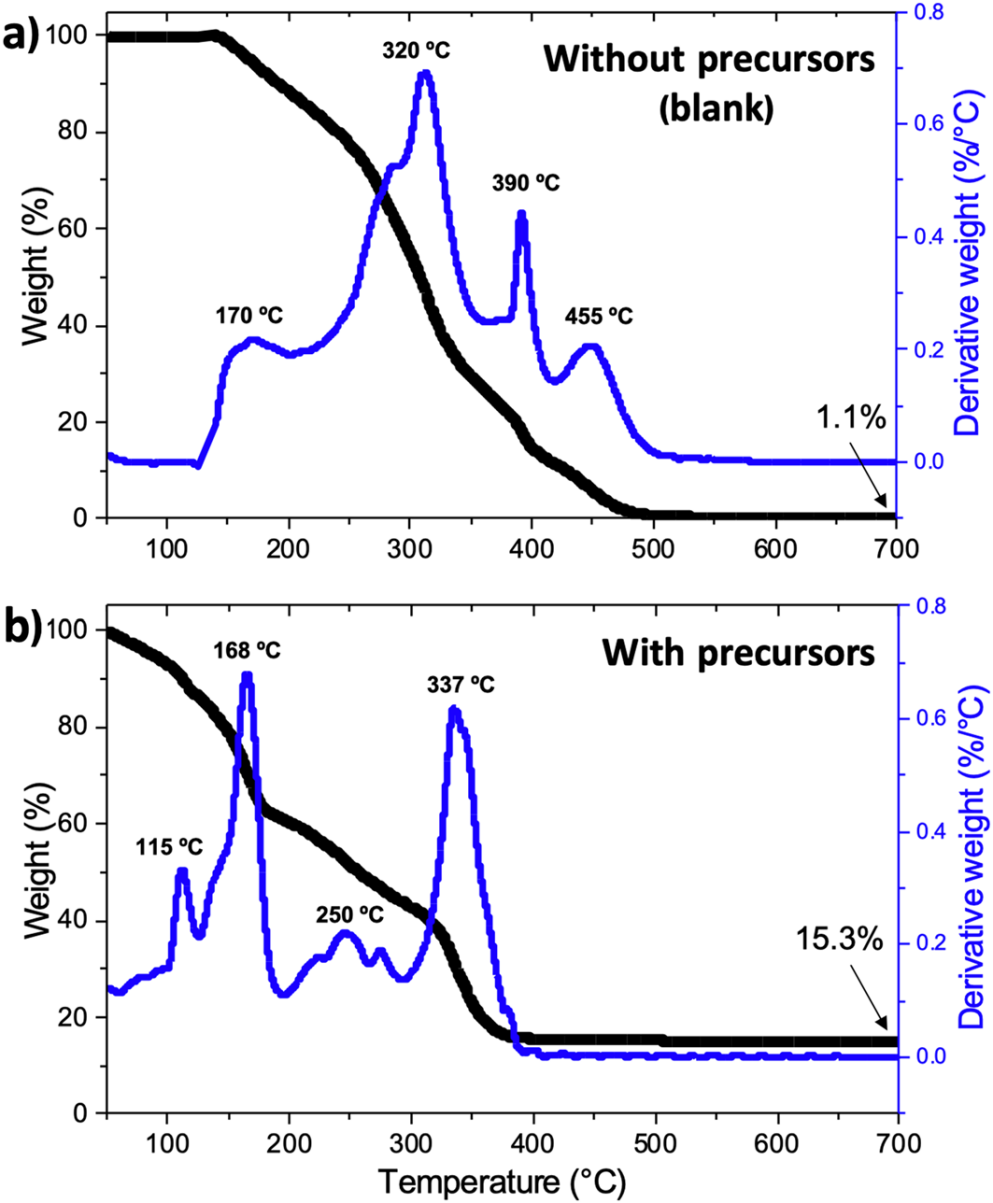
TG and DTG curves of UV-polymerized pieces from (**a**) blank resin without precursors, and (**b**) blank resin containing NMC precursors. The peak temperatures are only indicated for the major peaks.

The thermal post-processing is crucial for printed pieces, as choosing an appropriate thermal regime can significantly reduce defects formation that lead to mechanically-unstable structures^29^. Thermal decomposition profiles of two UV-polymerized pieces of blank resin and composite resin, respectively without and with precursor salts, were analyzed by TGA to determine debinding, synthesis, and sintering temperatures (Fig. 2a and b). The blank printed piece (which serves as a reference) composed of PEGDA polymer matrix, TPO photoinitiator and water showed 98.9% weight loss between 100 and 490 °C, and 99.8% by 560 °C (Fig. 2a). The TG curve of the blank printed piece also exhibits a first weight loss of 12% starting at 100 °C and ending right below 200 °C, with a DTG peak centered at 170 °C, which corresponds to water evaporation. Water evaporation extended to 200 °C because of its entrapment within the crosslinked polymer matrix created during UV-polymerization. The volume ratio PEGDA:H_2_O 1:2.5 is not consistent with this first weight loss because most of the water is lost during 3D printing and cleaning of the printed pieces. Subsequently, a significant weight loss of about 76% was detected between 200 and 420 °C, with a DTG peak centered at 320 °C and 390 °C, corresponding mainly to the calcination of cross-linked PEGDA^30^. The small peak that can be seen centered at 285 °C is probably due to the contribution of the calcination of uncured liquid monomer and photoinitiator molecules. Lastly, around 12% weight loss is registered between 420 and 490 °C, which corresponds to the calcination of the remaining PEGDA backbone chain, cumulatively amounting to 98.9% of weight loss.

The presence of NMC precursors made a significant change in the thermal decomposition of this printed piece, as previously reported in thermal studies of composite PEGDA resins^29,31^. Figure 2b shows that the primary thermal decomposition occurred between 40 and 400 °C, leaving only 15.3% of residues, in good agreement with what was expected from the synthesis reaction of NMC 111 according to the following stoichiometric Eq. (1).

1 $$\begin{gathered} {\text{3LiNO}}_{{3}} + {\text{ Ni}}\left( {{\text{NO}}_{{3}} } \right)_{{2}} .{\text{9H}}_{{2}} {\text{O }} + {\text{ Mn}}\left( {{\text{NO}}_{{3}} } \right)_{{2}} .{\text{4H}}_{{2}} {\text{O }} + {\text{ Co}}\left( {{\text{NO}}_{{3}} } \right)_{{2}} .{\text{6H}}_{{2}} {\text{O }} + n_{PEGDA} {\text{C}}_{{{26}}} {\text{H}}_{{{46}}} {\text{O}}_{{{13}}} + \, ({31}n_{PEGDA} - \hfill \\ {1}.{5}){\text{O}}_{{2}} \to {\text{3LiNi}}_{{{1}/{3}}} {\text{Mn}}_{{{1}/{3}}} {\text{Co}}_{{{1}/{3}}} {\text{O}}_{{2}} + {\text{ 9NO}}_{{2}} + \, \left( {{23}n_{PEGDA} + { 16}} \right){\text{H}}_{{2}} {\text{O }} + { 26}n_{PEGDA} {\text{CO}}_{{2}} \hfill \\ \end{gathered}$$

The first weight loss of around 15% between 50 and 125 °C with a DTG peak centered at 115 °C was attributed to water evaporation. Due to the presence of a high concentration of precursor salts, water evaporates more easily from the swollen PEGDA that presents elongated cross-linking bonds than from the tight-knit mesh produced in the blank printed piece^32^. This process occurs up to 200 °C, at the same time that trapped water and bound water from the nitrate salts is evaporated, and uncured monomer and photoinitiator molecules are calcined^33^. Since the sharp DTG peak centered at 168 °C represents bigger mass loss than for the blank piece, it is presumed that the synthesis of NMC 111 is also taking place at this temperature. Further evidence of the synthesis of NMC 111 from the nitrate salts is given by the multiple DTG peaks observed between 200 and 300 °C, which fits very well with the thermal decomposition of nitrate salts^33,34^. Just as for the blank printed piece, a prominent peak corresponding to PEGDA decomposition appears above 300 °C, and surprisingly, the whole process ends at 400 °C, at least one hundred degrees less than for the blank. The synthesis of NMC 111 profits from PEGDA decomposition to complete its synthesis, as recently proposed by Yee et al.^19^ and as presented in Eq. (1). Another possibility is that the metal nitrate salts catalyze PEGDA combustion, as reported before for organic matter^35,36^.

**Figure 3:**
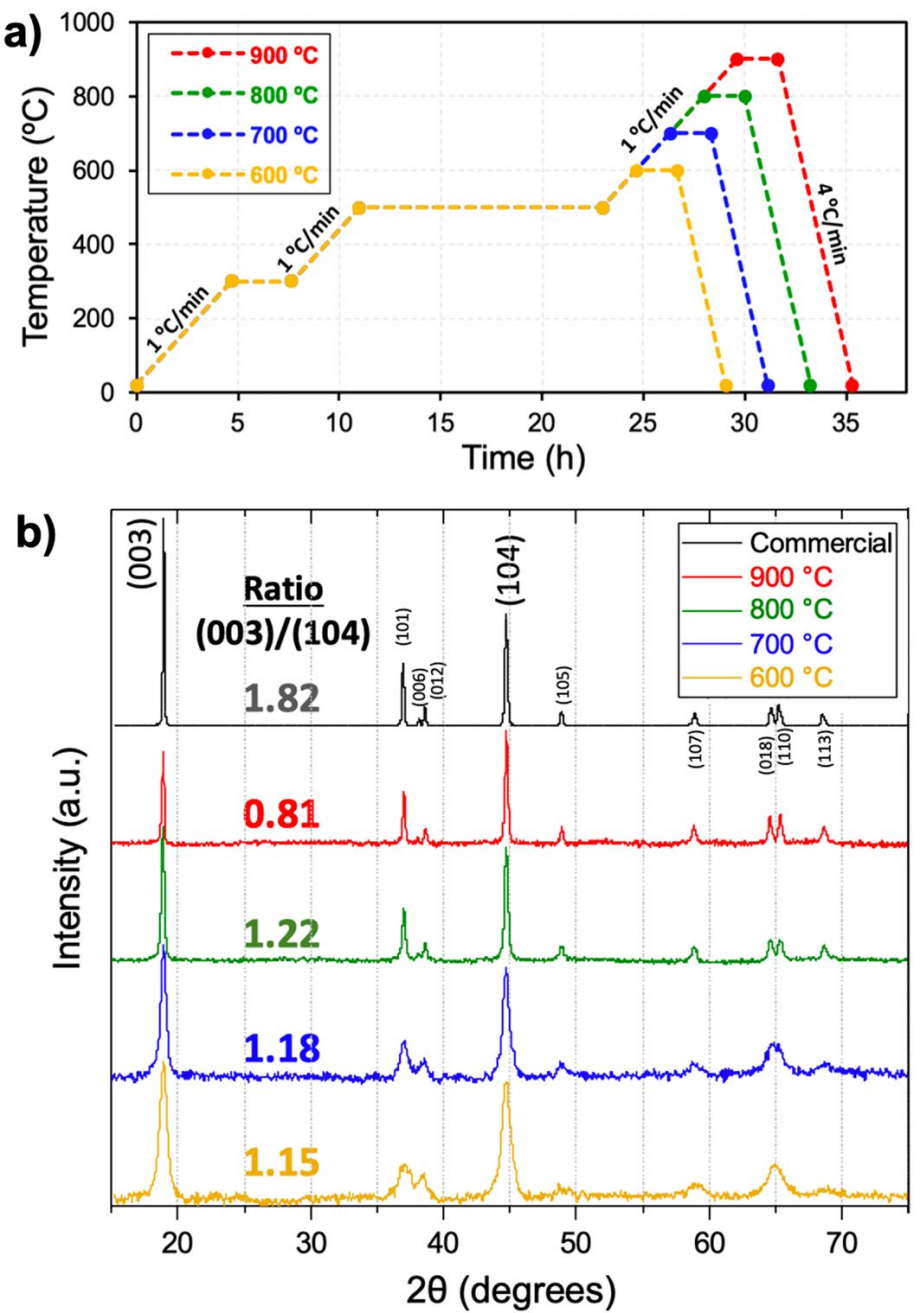
(**a**) Temperature profile used to synthesize NMC 111 from the composite polymer matrix. (**b**) XRD diffractograms of the four NMC 111 materials synthesized at a maximum temperature of 600, 700, 800 and 900 °C, and a commercial sample. The written ratios correspond to the intensity ratio of reflections (003)/(104).

Based on these TGA results, a first thermal post-processing profile was defined (Fig. 3a). The debinding step consisted of a heating ramp at 1 °C min^−1^ from room temperature to 500 °C (held for 12 h), with an intermediary plateau at 300 °C for 3 h to allow efficient water removal. Then, a high temperature step was implemented to allow the crystallization of NMC 111, analogous to a sintering step. The whole process was done under flowing air since the synthesis of NMC mostly relies on oxygen and lithium migration to crystallize the correct phase^37^. Four different sintering temperatures between 600 and 900 °C were studied to determine its impact on the electrochemical performance and morphology (Fig. 3b). Serving here as reference, a commercial NMC 111 was also analyzed, and to ensure a good comparison, all XRD diffractograms were normalized by the peak centered at 44.7 degrees (Fig. S1). All of the materials exhibit a layered structure based on the hexagonal ⍺-NaFeO_2_ structure with R-3m crystal group, typical of layered NMC materials^38^. The XRD analysis also corroborated that other significant phases of transition metals with other oxidation states were not present in the bulk of the material. This information however does not eliminate the possibility that traces of metal oxides exist on the surface of the materials. Moreover, the materials synthesized at 700, 800 and 900 °C present splitting into doublets the reflections (006)–(012) and (018)–(110), similar to the commercial sample, which has been reported to be a sign of good crystallinity^39^. Further, the I(003)/I(104) ratio was calculated for the five samples, understanding that ratio values above 1.20 denote low cation mixing between Li^+^ (0.76 Å) and Ni^2+^ (0.69 Å)^39,40^. The commercial NMC sample displays a high I(003)/I(104) ratio of 1.82. In contrast, the 900 °C sample exhibits a comparatively low ratio of 0.81, most probably due to lithium deficiency as a consequence of lithium volatilization. The closest intensity ratio to the commercial sample was observed for the 700 and 800 °C samples exhibiting ratios of 1.18 and 1.22, respectively. Therefore, these samples are expected to exhibit the best electrochemical performance. Although the 600 °C sample exhibits the main peaks corresponding to the correct NMC 111 crystalline phase, it displays a ratio of 1.15 and the doublet reflections are almost absent, thus indicating that this temperature was too low to crystallize NMC.

### Impact of temperature on the elemental composition, microstructure, and electrochemical performances

Along with the crystallinity analysis, the four samples were digested in *aqua regia* and analyzed by ICP-OES in order to confirm the molar composition of lithium, nickel, manganese and cobalt (Table 1). These analyses confirmed the composition to be close to the expected LiNi_1/3_Mn_1/3_Co_1/3_O_2_, in good agreement with the stoichiometric amount of precursor salts initially added to the resin. Interestingly, the molar amount of manganese and cobalt is almost constant regardless of the sintering temperature, whereas the molar amount of lithium decreases when the sintering temperature increases. The latter corroborates lithium volatilization in the form of Li_2_O that is expected to occur above 800 °C in the presence of oxygen^41^, even if 5% excess of lithium was added to compensate for this loss. As to the molar amount of nickel that varies between 0.30 and 0.33 mmol, it may be explained by the formation of metal oxides that deposited onto the crucible, rather than an error in the starting solution (identical for every sample).

**Table 1:** ICP-OES results of the four synthesized NMC 111.

Synthesis temperature	Elemental composition[mmol ± 0.01]			
	Li	Ni	Mn	Co
900 °C	0.97	0.32	0.32	0.30
800 °C	0.98	0.30	0.32	0.29
700 °C	1.00	0.33	0.32	0.30
600 °C	1.02	0.32	0.32	0.30

**Figure 4:**
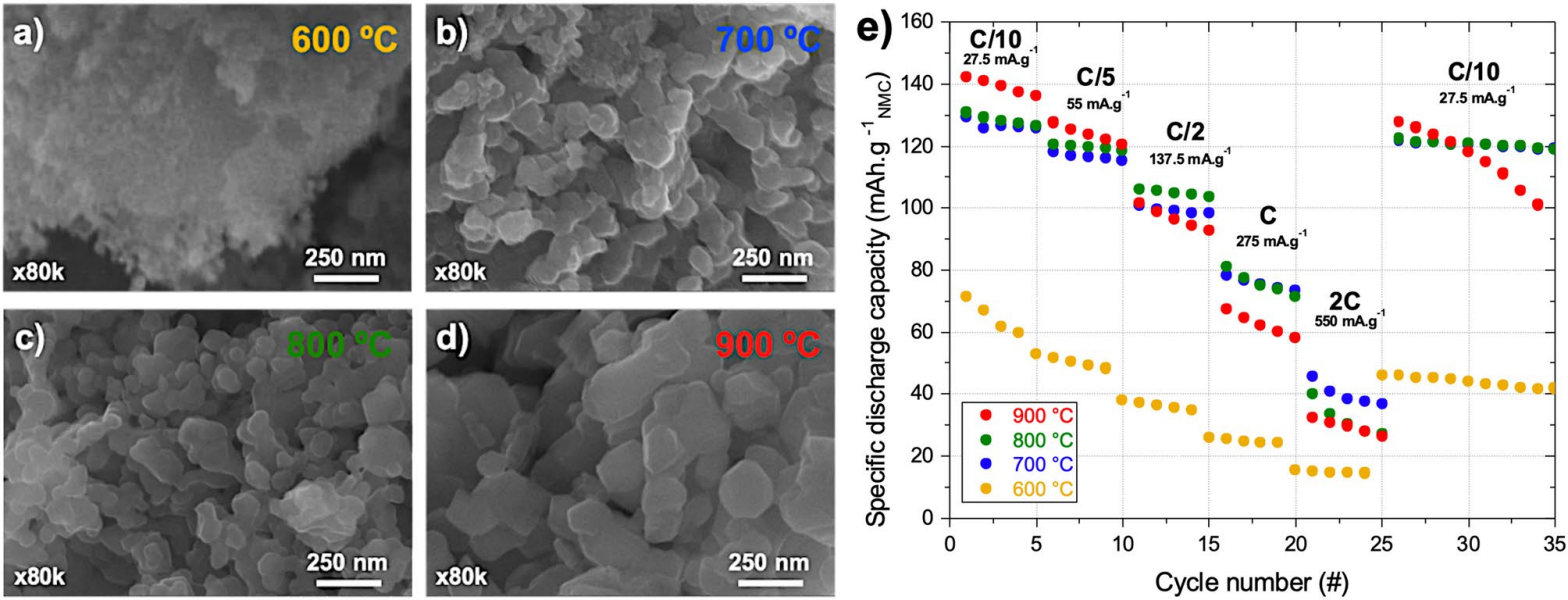
(**a-d**) Representative SEM micrographs of the NMC 111 materials synthesized at four different maximum temperatures. (**e**) Rate capability tests results for the four NMC 111 materials.

The impact of the sintering temperature on the microstructure was then studied using a Scanning Electron Microscope (SEM) (Fig. 4a-d). Interestingly, none of the four NMC 111 samples exhibited micro-sized granular secondary particles composed of submicron primary particles, as typically observed for NMC materials^38^. Instead, only NMC nanoparticles were observed. Such occurrence can be explained by the presence of the polymeric matrix, which could have prevented growth of secondary particles, similar to what occurs during molten flux synthesis^42^ and solid–liquid rheological reactions typically used to produce single-crystal NMC^43^. Increasing the precursor concentrations in the resin would decrease the distance for mass transport and lead to faster particle growth. Still, it would also mean less polymeric matrix and thus printability issues. As temperature increased from 600 to 900 °C, the average particle sizes increased also increased from < 50 nm, 90–140 nm, 100–180 nm, and 150–200 nm for NMC 111 synthesized at 600, 700, 800, and 900 °C, respectively. Finally, since particle sizes are few tens of nanometers, the sample prepared at 600 °C may be susceptible to surface side reactions upon cycling, which is expected to result in poor electrochemical performances^44^.

The impact of the synthesis temperature on the electrochemical performances was evaluated in the form of powder through rate capability tests (Fig. 4e). During the initial cycles, the 900 °C sample exhibits the highest specific discharge capacity with values of 137 and 122 mAh g^−1^, respectively, after 5 cycles at C/10 and C/5. After 5 cycles at the higher C-rates of C/2, 1C and 2C, the electrochemical performance however quickly degraded to 93, 59 and 28 mAh g^−1^, exhibiting even lower capacity values than the 700 and 800 °C samples. Furthermore, the 900 °C sample exhibits poor capacity retention when coming back at C/10 after 34th cycles. As predicted from the SEM images and XRD analysis, the sample synthesized at 600 °C exhibits a lower capacity retention than the other samples at every C-rate (only 60 mAh g^−1^ after 4 cycles at C/10, followed by values of 50, 36, 24 and 15 mAh g^−1^ after 5 cycles at C/5, C/2, 1C and 2C respectively), caused by detrimental surface side reactions related to its nanometric particle size and poor crystallinity. Another explanation might be related to the loss of electronic conductivity, as it is easier for smaller active material particles to become electrically isolated upon cycling^45^. On the contrary, the 700 °C and 800 °C samples present a more stable performance upon cycling. Both samples actually exhibit very similar specific discharge capacity values at the different studied C-rates: 126, 116, 99, 75 and 38 mAh g^−1^ after 5 cycles at C/10, C/5, C/2, 1C and 2C respectively for the sample synthesized at 700 °C; and 127, 119, 104, 71 and 30 mAh g^−1^ after 5 cycles at C/10, C/5, C/2, 1C and 2C respectively for the sample synthesized at 800 °C. When coming back at C/10, good capacity retention is observed for both samples during 10 cycles. Noteworthy, the initial specific capacity values are comparable to those reported in the literature of NMC 111 synthesized from nitrate salts of 140–155 mAh g^−1^ and analyzed under the potential window indicated in this work^46,47^.

Moreover, the derivative dQ/dV curves of the first cycle (Fig. S2a) allowed to deduce that the samples synthesized at 800 and 900 °C present only one clearly defined reversible electrochemical process with maximum activity between 3.78 and 3.8 V *vs*. Li/Li^+^ (delithiation) and 3.72 V (lithiation), corresponding to the structural transition from 1^st^ hexagonal to monoclinic phase upon lithium deintercalation^48^. Intriguingly, the sample synthesized at 700 °C presents a second reversible electrochemical process with maximum activity at 3.93 V (delithiation) and 3.88 V (lithiation). This process occurring at a higher potential corresponds to transitions between states in which lithium is ordered and disordered depending on the available octahedral or tetrahedral vacant sites in the crystal structure^38^, and it is possibly present due to the lower sintering temperature that reduced lithium volatilization, thus allowing lithium ions transitions. The latter assumption is in line with the observations of the 600 °C sample, which exhibits very faintly both processes. The potential gap between delithiation and lithiation in all samples not exceeding 0.1 V can be regarded as normal for NMC materials, and is attributed to internal polarization.

### Optimization of the heating rate during the debinding step

**Figure 5:**
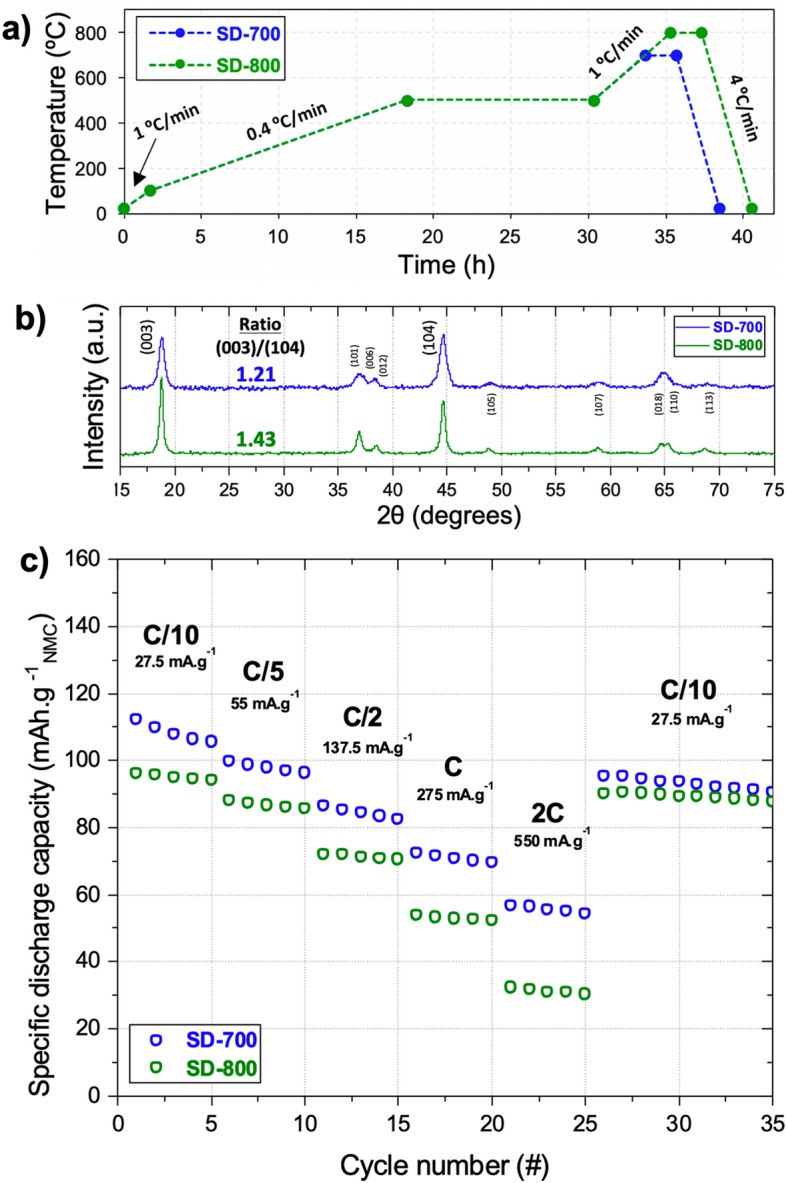
(**a**) Temperature profile to synthesize NMC 111 from two 3D printed items including a slower heating rate step and synthesized at a maximum temperature of 700 or 800 °C. (**b**) XRD diffractograms of samples SD-700 and SD-800 NMC 111 materials. The peak normalization is shown in Fig. S4. (**c**) Rate capability tests results for the two NMC 111 materials.

These results are a testament to the strong impact of the temperature profile on the resulting electrochemical performances of NMC 111 as positive electrode material. Based only on these results, both NMC 111 sintered at 700 and 800 °C delivered the best specific capacities among the four. It is however important to mention that these four 3D NMC electrodes were brittle, as structures tend to collapse upon handling. For this reason, the next stage of this work was focused on optimizing the debinding rate with a view to ensure structural integrity, while maintaining as high as possible electrochemical performance. The heating rate applied during the debinding step is particularly critical for VPP 3D printed items as it involves the removal of all electrochemically inactive components, such as the polymer matrix and photoinitiator. As shown in Eq. (1), the polymer matrix (PEGDA) is indeed thermally decomposed into CO_2_ and H_2_O gasses, which may generate cracks and deformations of the 3D item, thus compromising the mechanical integrity (observed by SEM and is illustrated in Fig. S3a and b). As reported in literature^49^, applying a slower heating rate during the debinding step would considerably reduce cracks and deformations, as a longer time is left for gasses release. In this context, two additional samples were synthesized. A common debinding step was implemented for both samples (Fig. 5a), which consisted of a first heating ramp at 1 °C min^−1^ from room temperature to 100 °C, followed by a slower heating ramp at 0.4 °C min^−1^ up to 500 °C (held during 12 h). Finally, samples were heated to 700 °C or 800 °C at 1 °C min^−1^ and the temperature was held for 2 h (unchanged condition). Hereafter, samples obtained with a slow debinding (SD) are referred to as SD-700 °C and SD-800 °C. XRD and ICP-OES analyses revealed that the molar ratio was similar to previous samples and the crystalline structure was preserved (Fig. 5b and Table S1). Nonetheless, the SD-800 °C sample lost its clearly defined doublets (006)-(012) and (018)-(110), compared to the previous 800ºC sample; whereas SD-700 °C maintained similar non-defined doublets. Both SD-700 °C and SD-800 °C present I(003)/I(104) ratios of 1.21 and 1.43, respectively, but broader peaks than those of the 700 °C and 800 °C samples. Peak broadening means either that the sample presents particles in the nanoscale or that lattice defects exist in large enough abundance^50^. Since it has been observed that increasing the temperature leads to particle growth, it is possible to attest that the peak broadening is rather due to induced lower crystallinity when introducing a slower debinding step. A more complete structural analysis must be achieved in a future study, but it was left out of the scope here, since this work is meant to rather emphasize the creation of complex-shaped NMC 111 electrodes via 3D printing.

From the electrochemical standpoint, the addition of a slower debinding step decreased the specific discharge capacities at every tested current density (Fig. 5c). Samples SD-700ºC and SD-800 °C respectively displayed a lower initial specific discharge capacity of 112 mAh g^−1^ (− 9.7%) and 98 mAh g^−1^ (− 21.6%), in comparison with their counterparts. A similar trend was observed after 35 cycles, when coming back at C/10, as samples SD-700 °C and SD-800 °C respectively displayed a lower specific discharge of 92 and 90 mAh·g^-1^, while their counterparts exhibited 120 and 119 mAh g^−1^. The lower discharge capacity values can be related to the longer exposure of the sample to heat (18 h instead of 11 h) that potentially leads to increased atom mobility and disorder in the earliest stages of atom organization. Based on the previous observation of the I(003)/I(104) ratio for both SD-700 °C and SD-800 °C samples, the latter was expected to exhibit better electrochemical performance. However, as described below, the intensity ratio comparison from diffractograms is a way to predict cation mixing, but it is certainly not enough to predict the final electrochemical performance. Indeed, as shown in the results from the rate capability tests in the form of powder (Fig. 5c), SD-700 °C presents higher specific discharge capacities (106, 97, 82, 71 and 57 mAh g^−1^ after 5 cycles at C/10, C/5, C/2, 1C and 2C, respectively) than the SD-800 °C sample (94, 85, 71, 55 and 31 mAh g^−1^ after 5 cycles at C/10, C/5, C/2, 1C and 2C, respectively). Nonetheless, after 35 cycles the capacity retention of SD-800 °C was 89%, in contrast to 82% for SD-700 °C. Although their structural integrity was improved, these two samples appeared still brittle. To overcome this issue, a faster heating rate during the sintering step was implemented (motivated by observations from previous studies^51^), added to the slow debinding step that was already introduced in this section.

### Compromise between mechanical integrity and electrochemical performance

**Figure 6:**
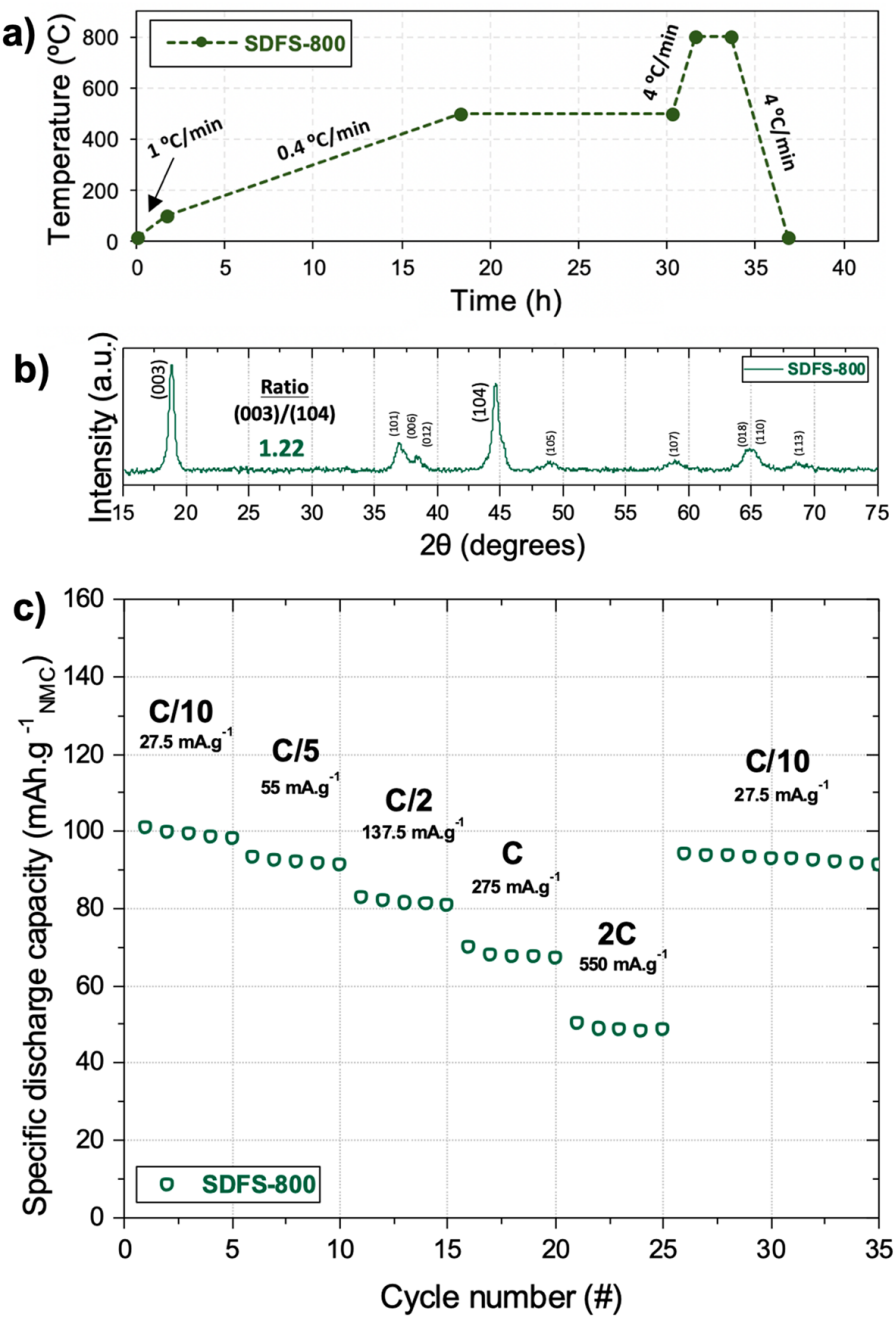
(**a**) Temperature profile including a slower heating rate step and a faster sintering step at a maximum temperature of 800 °C to synthesize NMC 111 from a 3D printed item. (**b**) XRD diffractogram of the synthesized SDFS-800 NMC 111 material. (**c**) Rate capability tests results of the SDFS-800 material.

**Figure 7:**
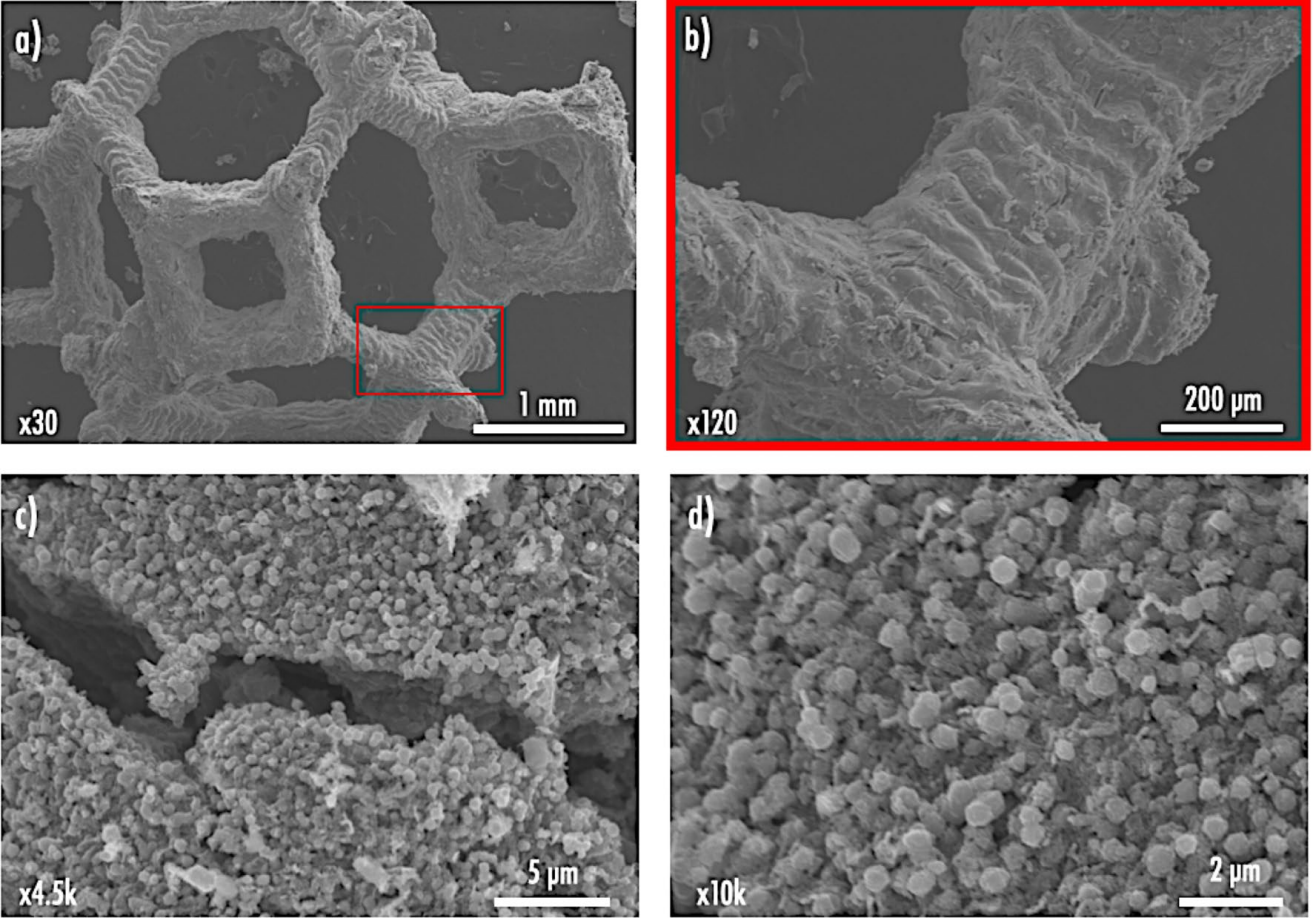
Representative SEM micrographs of different areas of the printed electrodes.

A last NMC 111 sample was synthesized from a 3D printed item subjected to a first heating ramp at 1 °C min^−1^ from room temperature to 100 °C, followed by a slower heating ramp at 0.4 C min^-1^ up to 500 °C (held during 12 h) for the debinding step (Fig. 6a). The sample then endured a faster heating rate of 4 °C min^−1^ from 500 to 800 °C (held for 2 h) for the sintering step. This sample, subjected to both slow debinding (SD) and fast sintering (FS) is called SDFS-800 °C. The faster heating rate to reach the synthesis temperature was added to promote the fusion of particles together in order to eliminate the porosity that was left after the debinding process^52^. This was shown to inherently improve the structural integrity as it was possible to handle the resulting sample SDFS-800 °C for further SEM imaging. As shown in Fig. 7a, complex 3D structures of NMC 111 battery materials were obtained by means of VPP precursors approach, thus paving the way for their integration in niche applications requiring shape-conformable energy storage devices. From the close-up SEM images, it can be seen that the 3D NMC 111 electrode still displays imperfections such as the printing layers (Fig. 7b) inherent to the additive manufacturing VPP process, and some cracks created during the thermal post-processing step (Fig. 7c). One remarkable difference regarding the particle size and morphology is that the primary particles aggregated much more than what was observed in Fig. 4c (compare to Fig. 7d). Previously, for the 800 °C sample the particles were found to be 100–180 nm, whereas the SDFS-800 °C sample presents secondary particles of about 500 nm formed from the aggregation of smaller primary particles during the faster sintering step.

When optimizing this innovative VPP 3D printing of NMC via the precursor approach, a compromise between electrochemical performance and mechanical properties is unavoidable. This is meant to counteract the high level of densification that is normally achieved upon sintering classical VPP printed parts, that could prevent the existence of sufficient porosity for electrolyte impregnation. In this work, it was shown that the SDFS-800 °C sample presents the best structural integrity among all samples, but at the expense of the electrochemical performance, as the obtained capacity values can still be further improved to previously reported values^38^. As shown in Fig. 6c, from the electrochemical standpoint, sample SDFS-800 °C exhibited specific discharge capacities of 98, 92, 81, 68 and 50 mAh g^−1^ after 5 cycles at C/10, C/5, C/2, 1C and 2C respectively). After 35 cycles, a capacity of 92 mAh g^−1^ was obtained at C/10, thus corresponding to a respectable capacity retention of 90%. While classical sintering processing of oxide ceramics that are meant to further improve the mechanical strength usually involves heating steps at temperatures well above 800 °C^38^, in this work those steps were avoided to prevent drastic grain growth and lithium volatilization that would have a detrimental effect on the electrochemical performance. Similar to the SD-800 °C sample, the XRD of SDFS-800 °C sample also shows the absence of defined (006)-(012) and (018)-(110) doublets, while still presenting broad peaks (Fig. 6b). The I(003)/I(104) ratio was calculated to be 1.22, lower than the 1.43 value observed for the previous SD-800ºC sample.

### Toward multi-material VPP additive manufacturing of a complete 3D battery

**Figure 8:**
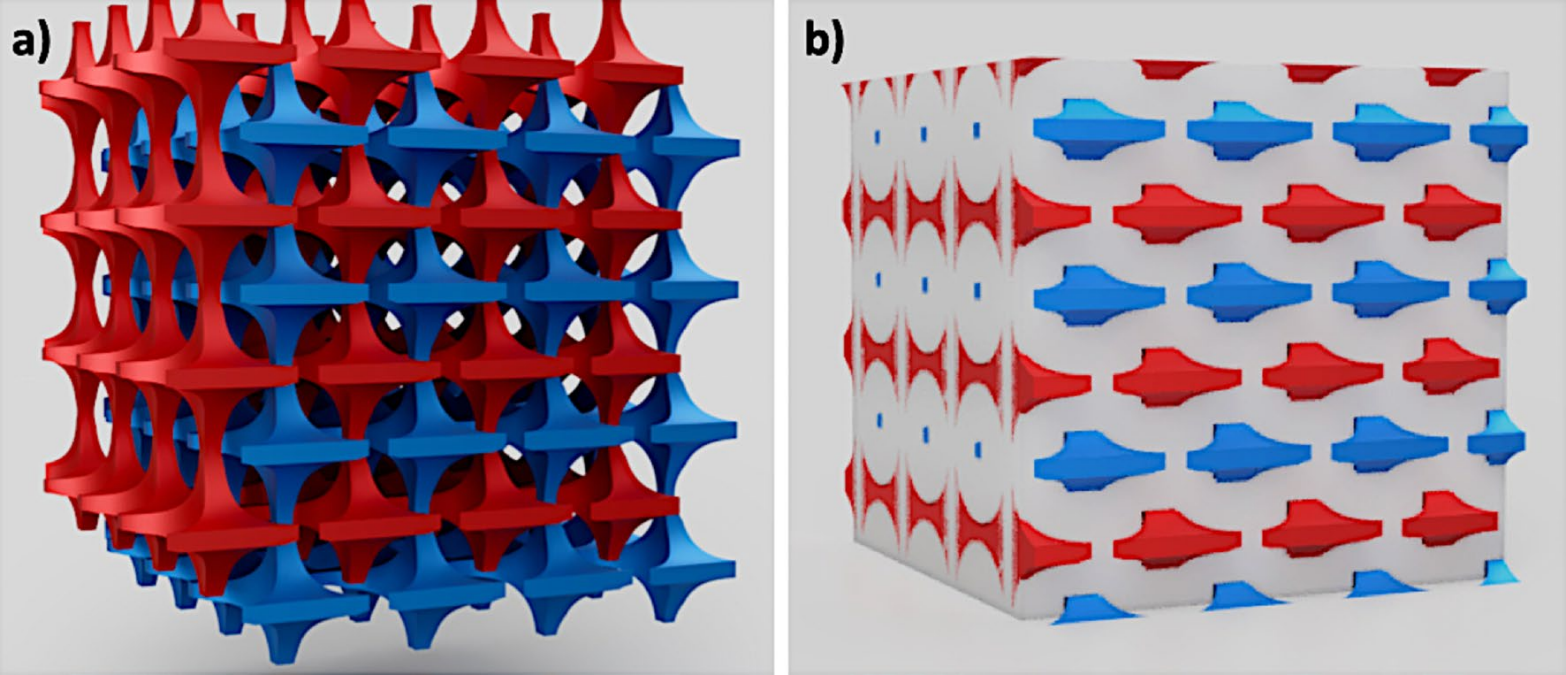
Schematic of a 3D battery architecture that could only be obtained thanks to multi-material printing options: (**a**) without, and (**b**) with the electrolyte displayed (blue: positive electrode, red: negative electrode, white: solid electrolyte).

Focused specifically on the NMC 111 material as a positive electrode, this work appears as the first stage towards the printability of a complete 3D lithium-ion battery in one single print (or “one-shot'”) via VPP. A thorough investigation on multi-material additive manufacturing is further required to demonstrate the printability of complex 3D battery designs (Fig. 8) that could lead to improved power performances^3,4,53,54^. While multi-material printing options^55,56^ had been already commercialized for material extrusion process^54^, only few experiments at the laboratory scale have been reported for VPP process^57–60^. Considering that precise multi-material printing of three different precursor resins (positive and negative electrode and solid ceramic electrolyte) is achievable, an important difficulty will still reside in the thermal postprocessing step as debinding and sintering temperatures must be tuned depending on the polymeric matrix and/or electroactive materials. Future research directions include the study of multi-material structures to improve overall mechanical performance in which one material such as a metallic current collector could serve as the load-bearing structure for structural batteries. In contrast, differences in coefficients of thermal expansion could aggravate delamination, particularly in the context of complex 3D geometries.

## Conclusion

For the first time, VPP 3D printing precursor approach was employed to synthesize complex 3D battery electrodes of NMC 111 ternary oxide material. This work showed that this innovative synthesis route is capable of producing battery electrodes with complex shapes and submicron features, while maintaining adequate electrochemical performance. As material feedstock for the VPP 3D printer, a novel aqueous composite photocurable resin loaded with soluble precursor salts was prepared. Complex 3D printed structures were then produced thanks to the minimization of the detrimental light scattering and increased viscosity given by this method. With a view to maintain the structural integrity of the 3D NMC 111 item while maximizing the electrochemical performance, a combination of slow heating rate (0.4 °C min^−1^) during the debinding step followed by a faster heating rate (4 °C min^−1^) during the sintering stage, were implemented. Specific discharge capacities of 98 mAh g^−1^, 92 mAh g^−1^, 81 mAh g^−1^, 68 mAh g^−1^ and 50 mAh g^−1^ after 5 cycles at C/10, C/5, C/2, 1C and 2C respectively, and a capacity retention of 90% after 35 cycles were obtained. Future work related to the precursor approach will be concentrated on the addition of conductive additives, and on leveraging the polymeric resin as a carbon source upon thermal post-processing. Other gaps that need to be addressed for mainstream use of this method are, to increase the amount of soluble salts within the resin (engineer the salts’ solubility by adding other additives), further study the impact of the oxygen concentration during thermal post-processing to favor crystallinity, and explore the impact of the printing parameters on cracks formation.

Finally, the innovative precursor approach explored in this work could be easily transposed to higher resolution additive manufacturing processes as it is particularly suitable for two-photon polymerization (at the expense of production sizes and rates) as well as inkjetting with an associated print-head-attached UV lamp (material jetting per ISO/ASTM 52,900 standard terminology), due to the low viscosity of the resin and absence of solid particles usually reported to have a detrimental impact on the printability due to UV light-scattering. No doubt that future years will witness the successful development of a complex 3D electrode structure containing both the electroactive material and conductive additives that will pave the way to fully functional 3D printed batteries.

## Materials and methods

### Composite resin formulation

Four independent 3 mol L^−1^ solutions of lithium nitrate (LiNO_3_, ReagentPlus Sigma Aldrich), nickel(II) nitrate hexahydrate (Ni(NO_3_)_2_.6H_2_O, ≥ 97% Sigma Aldrich), cobalt(II) nitrate hexahydrate (Co(NO_3_)_2_.6H_2_O, ≥ 98% Sigma Aldrich), and manganese(II) nitrate tetrahydrate (Mn(NO_3_)_2_.4H_2_O, ≥ 97% Sigma Aldrich) were prepared and combined in stoichiometric amounts to yield NMC 111 upon synthesis. Lower solution concentrations were tested but did not produce as good result as the one reported in this work. Only LiNO_3_ was added in excess of 5 mol% to compensate for lithium volatilization at high-temperature^41^. In a separate container, poly(ethylene glycol) diacrylate (PEGDA avg. Mn 575, (C_2_H_4_O)_n_C_6_H_6_O_3_, Sigma Aldrich) and diphenyl(2,4,6-trimethylbenzoyl) phosphine oxide (TPO, 97%, Sigma Aldrich) were combined in a mass ratio PEGDA:TPO of 1:0.002 and magnetically stirred for 30 min. Finally, the photopolymerizable resin was prepared by combining both solution mixtures in a ratio PEGDA:precursor solution of 1:3.33 vol. and stirred for another 30 min. The resulting composite resin was transferred onto a UV filtering container and kept at 10 °C until used for 3D printing.

### Vat photopolymerization 3D printing

Three dimensional models were designed with Fusion 360 (Autodesk, USA) and nTopology softwares (nTopology, USA). The architectures consist of six discs (16 mm diameter and 3 mm thick) exhibiting various infill patterns (octet, fluorite, splitP, cylindrical gyroid, classical gyroid, and diamond) with 30% infill density, as well as a 20 mm^3^ cube composed of 64 units of a truncated octahedron (14 faces, 8 regular hexagons and 6 squares) or faujasite infill-type lattice (shown in Fig. 1b). Both discs and cube exhibit beams in the 0.5 mm to 0.8 mm thickness range. The models in Standard Tessellation Language (.stl files) were sliced into 50 μm thick 2D-slices using Tethonware software (Tethon 3D, USA). Printing of the 3D structures was performed at 25 °C using a Bison 1000 Digital Light Processing 3D printer (Tethon 3D, USA) after loading 150 mL of the composite resin. To ensure good printing quality, an initial light exposure time of at least 80 s was set for the 10 first printed layers. Subsequent layers were subjected to light exposure for at least 50 s. Prior to printing, the bed platform and tank were methodically cleaned with 2-propanol while the afore-prepared composite resin was stirred for 30 min.

### Thermal post-processing of printed items

The 3D printed items were placed on a quartz boat and introduced inside a GSL-1700X tubular furnace (MTI Corporation, USA) for thermal treatment. The latter consisted in a debinding step to remove the polymer matrix^56^, followed by a sintering step where the in-situ synthesis of NMC 111 material occurred. Both steps were performed under flowing air at a pressure of 0.025 MPa. The thermal profiles that were employed can be found in the *Results and discussion* section.

### Materials characterization

Thermogravimetric analysis (TGA) was executed by means of a Q50 (TA instrument). Between 7 and 11 mg of material was placed on a platinum pan and analyzed between 25 and 900 °C at a heating rate of 1 K min^−1^ oxygen as sample gas at 45.0 mL min^−1^. Total lithium, nickel, manganese and cobalt molar content in the NMC samples were determined using an Inductively coupled plasma optical emission spectrometer (ICP-OES) (Perkin-Elmer Optima 4300 DV, Shelton, CT). Approximately 25–50 mg of samples were acid digested using 5 mL of a mixture of concentrated trace pure HNO_3_ and HCl (1:3) at 115 °C for 45 min in a Digiprep hot-block (SCP Science, Canada). The ICP-OES parameters used were as follows: nebulizer flow, 0.80 L min^−1^; power, 1,450 W; peristaltic pump rate, 1.5 mL min^−1^; flush time, 15 s; delay time, 20 s; read time, 10 s; and wash time, 60 s. Every sample was analyzed in triplicate. For quality control, a blank and a 1 mg L^−1^ standard solution of the analytes were analyzed every three NMC samples, with recoveries at or above 97%.

X-ray powder diffraction (XRD) diffractograms were acquired with an Empyrean-2 X-ray diffractometer (Malvern Panalytical, UK) using Cu Kα radiation (λ = 1.5418 Å). An accelerating voltage of 45 kV and a current of 40 mA were set to identify the crystalline structure of each powder. Data were obtained in the 2*theta* range from 10 to 90° with a step size of 0.013° and scan rate of 8° min^−1^.

The influence of the sintering temperature on particle size as well as material dispersion, sample homogeneity and presence of eventual cracks on the sintered 3D structures were investigated by means of a S-4800 (Hitachi, Japan) field emission scanning electron microscope (SEM) operating in high vacuum mode. Secondary images were recorded with a 15 kV acceleration voltage.

### Electrochemical characterization

NMC 111 structures synthesized after thermal post-processing were thoroughly mixed in a mortar with conductive carbon black Timcal Super C45 (BET = 45 m^2^ g^−1^ and 20 nm particle size, MSE Supplies) in a 9:1 weight ratio to improve the electronic conductivity. Swagelok-type half-cells were assembled inside an argon-filled glovebox (H_2_O < 0.1 ppm, O_2_ < 0.1 ppm). Lithium metal (0.38 mm thick ribbon, 99.9% purity, Sigma Aldrich) was used as a counter/reference electrode for half-cells, while 12 ± 0.2 mg of the NMC/C45 composite samples were employed as working electrode material. Fiberglass separator (Whatman GE Healthcare) was impregnated with 150 µL of 1 M LiPF_6_ in ethylene carbonate and dimethyl carbonate (EC:DMC 1:1 weight ratio) liquid electrolyte supplied by Sigma Aldrich. Prior cycling, the cells were left to rest for 6 h to allow complete electrolyte impregnation. Cells were galvanostatically charged and discharged at C/10 (27.5 mA g^−1^), C/5 (55 mA g^−1^), C/2 (137.5 mA g^−1^), 1C (275 mA g^−1^), 2C (550 mA g^−1^) and again at C/10, in a potential window of 2.5—4.3 V vs Li/Li^+^ and at 25 °C by means of a LBT galvanostat (Arbin Instruments, USA). The current densities were calculated by considering total extraction of lithium (x = 1 in Li_1-x_Ni_1/3_Mn_1/3_Co_1/3_O_2_).

## Supplementary Information

Supplementary Information.

## Data Availability

The data that support the findings of this study are available from the corresponding author upon reasonable request.
