# Supplementary material for: Additive manufacturing of LiNi1/3Mn1/3Co1/3O2 battery electrode material via vat photopolymerization precursor approach
Source: Sci Rep. 2022 Nov 8;12:19010. doi: 10.1038/s41598-022-22444-1 (PMC9643428; doi:10.1038/s41598-022-22444-1)
Supplement: Supplementary file 1 — Supplementary Information. [file 41598_2022_22444_MOESM1_ESM.docx]

**Supplementary information**

**Additive Manufacturing of LiNi_1/3_Mn_1/3_Co_1/3_O_2_ battery electrode material via vat photopolymerization precursor approach**

*Ana C. Martinez*, Alexis Maurel*, Ana P. Aranzola, Sylvie Grugeon, Stéphane Panier, Loic Dupont, Jose A. Hernandez-Viezcas, Bhargavi Mummareddy, Beth L. Armstrong, Pedro Cortes, Sreeprasad T. Sreenivasan*, and Eric MacDonald**


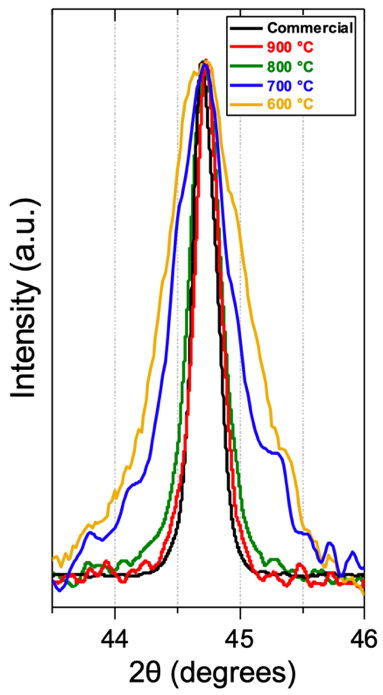


**Figure S1.** Diffractogram normalization of the commercial, 900 ºC, 800 ºC, 700 ºC, and 600 ºC samples by the (104) reflection centered at 44.7 degrees.


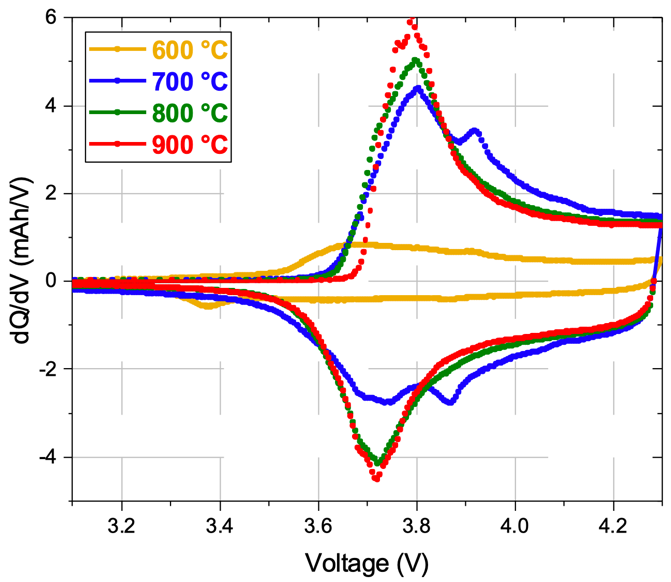


**Figure S2.** Derivative dQ/dV curves of the first cycle for the NMC 111 materials synthesized with maximum temperatures of 600ºC, 700ºC, 800ºC and 900ºC.


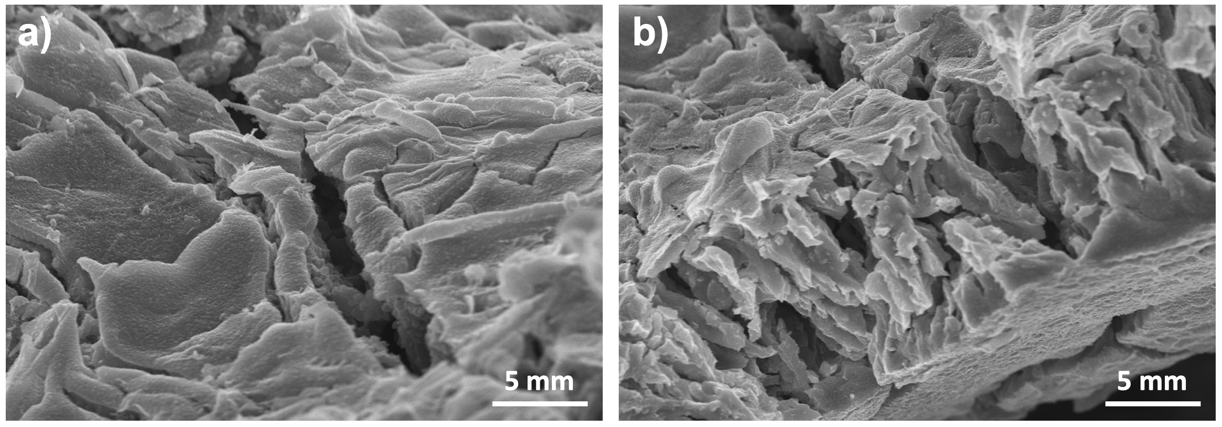


**Figure S3. a)** Frontal and **b)** lateral view of a printed structure of NMC 111 (synthesized at 800ºC), to explain and illustrate the brittleness of the sample.


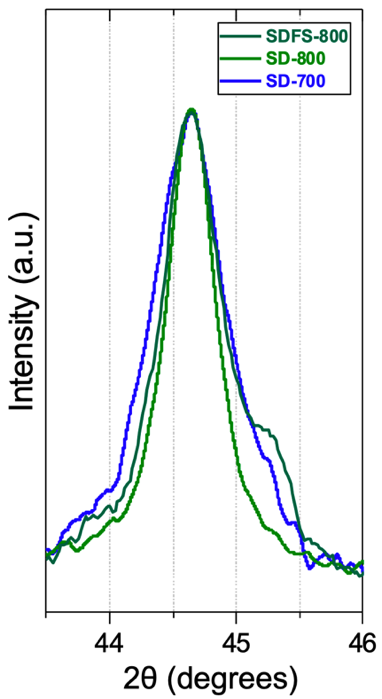


**Figure S4.** Diffractogram normalization of the commercial, SDFS-800, SD-800, and SD-700 samples by the (104) reflection centered at 44.7 degrees.

**Table S1.** ICP-OES results of three synthesized NMC 111.

| **Synthesis temperature** | **Elemental composition [mmol ± 0.01]** | | | |
| --- | --- | --- | --- | --- |
|  | **Li** | **Ni** | **Mn** | **Co** |
| **SD-700 ºC** | 1.01 | 0.32 | 0.33 | 0.29 |
| **SD-800 ºC** | 1.00 | 0.32 | 0.32 | 0.30 |
| **SDFS-800 ºC** | 0.94 | 0.33 | 0.33 | 0.27 |
